# The optic nerve sheath diameter on ultrasound can only be used as a marker of intracranial pressure after comparison with a gold standard

**DOI:** 10.1590/1806-9282.20260873

**Published:** 2026-09-21

**Authors:** Carla Alexandre Scorza, Fulvio Alexandre Scorza, Josef Finsterer

**Affiliations:** 1 Universidade Federal de São Paulo, Escola Paulista de Medicina – São Paulo (SP), Brazil; 2 Neurology and Neurophysiology Center – Vienna, Austria

Dear Editor,

We read with interest the article by Çelik et al. on the validity of measuring the optic nerve sheath diameter (ONSD) using transorbital ultrasound to assess intracranial pressure (ICP) in 50 patients with suspected elevated ICP^1^. The ONSD was higher in the patients than in the control group and correlated with mortality as well as with scores such as the Glasgow Coma Scale, Full Outline of Unresponsiveness, Acute Physiology and Chronic Health Evaluation II, and Sequential Organ Failure Assessment^1^. It was concluded that ONSD measurement is a reliable method for identifying patients with elevated ICP^1^. The study is interesting, but some points require further clarification.

The first point is that the conclusions drawn should be viewed with caution, as the ultrasound results were not compared to a gold standard for determining ICP^1^. The gold standard for measuring ICP in the intensive care unit is an ICP probe inserted through a burr hole^2^. Cerebral computed tomography and clinical presentation are not sufficient standards for assessing ICP. As long as the assessment of ICP via ONSD measurement is not compared to the gold standard, it remains uncertain whether ONSD measurement actually reflects elevated ICP.

Second, ONSD depends not only on ICP but also on several other factors^3^. These include age, gender, posture, the collagen and elastin content of the sclera and the optic nerve sheath, the stiffness of the surrounding tissue, intraocular pressure, intraorbital venous congestion, intraorbital pressure, intraorbital geometry, the morphology of the optic nerve, the morphology of the eyeball (curvature, radius, scleral thickness), blood pressure, respiratory status, altitude, coughing, upper body position, and currently taken medications^4^. Medications that increase ICP include vasodilators, opioids, vitamin A, tetracyclines, and lithium, while osmotic agents, diuretics, and some steroids can lower ICP^5^. As long as these confounding factors are not accounted for and the study cohort is not homogenized with respect to these confounders, the reliability of ONSD measurements using ultrasound remains low.

The third point is that it was not specified whether the measurements were performed by a single physician or by multiple physicians. If multiple physicians collected the data, interobserver variability should be calculated. Knowledge of interobserver variability is crucial, as it can vary significantly among different researchers.

The fourth point is that diurnal fluctuations in ICP were not taken into account^1^. Since ICP rises overnight during sleep (particularly during the REM phase), it is important to know whether the ONSD measurements were all taken at the same time and whether all measurements were performed on awake patients or not.

Overall, ONSD measurement remains unreliable as a substitute for ICP as long as not all determinants of ICP are taken into account. If not all determinants of ONSD are considered, this can lead to low sensitivity and specificity of the ONSD measurement, regardless of the technique used.

## Data Availability

The datasets generated and/or analyzed during the current study are available from the corresponding author upon reasonable request.
